# Pulmonary lymphangitic carcinomatosis as a primary manifestation of gastric carcinoma in a young adult: a case report and review of the literature

**DOI:** 10.1186/1756-0500-5-638

**Published:** 2012-11-16

**Authors:** Kim Moubax, Wim Wuyts, Vincent Vandecaveye, Hans Prenen

**Affiliations:** 1 Department of Gastroenterology, University Hospitals Leuven, Digestive Oncology Unit, Herestraat 49, 3000, Leuven, Belgium; 2 Department of Pneumology, University Hospitals Leuven, Leuven, Belgium; 3 Department of Radiology, University Hospitals Leuven, Leuven, Belgium

**Keywords:** Lymphangitic carcinomatosis, Gastric cancer, Whole body MRI

## Abstract

**Background:**

Lymphangitic carcinomatosis as a manifestation of gastric carcinoma is rare. The presenting symptoms are misleading and nonspecific, often resulting in delayed diagnosis.

**Case presentation:**

We present a case of a 24 year old male with progressive dyspnea. Initial radiologic assessment suggested interstitial lung disease, which was subsequently treated with antibiotics and corticosteroids. However, endoscopy and whole body diffusion-weighted magnetic resonance imaging revealed a metastatic gastric cancer with the presence of lymphangitic carcinomatosis.

**Conclusions:**

Pulmonary lymphangitic carcinomatosis is a rare manifestation of metastatic gastric cancer. Patients present with severe but non-specific respiratory complaints. Definitive diagnosis can be achieved by transbronchial biopsy. Prognosis is poor and optimal treatment is not defined. Whole body diffusion-weighted magnetic resonance imaging is a promising imaging tool for the diagnosis of metastatic gastric cancer.

## Background

Pulmonary lymphangitic carcinomatosis is present in 6-8% of patients with lung metastases [1]. Infiltration of the pleural, peribronchial and perivascular lymphatics by neoplastic cells was first noted in 1829. The histopathology was described in detail in 1874. The spread of tumour cells to the pulmonary lymphatic system or the adjacent interstitial tissue results in thickening of the bronchovascular bundles and septa. Desmoplastic reaction, due to proliferation of neoplastic cells and lymphatic dilatation by edema fluid or tumour secretions contribute to this interstitial thickening [2]. Spread of the neoplasm outside the interstitium and lymphatic spaces into the adjacent parenchyma can result in a nodular pattern [2].

Herein we report a very rare case of lymphangitic carcinomatosis as a primary manifestation of gastric carcinoma in a young adult.

## Case Presentation

A 24 year old man presented at the emergency department after an episode of hemoptysis. For six months he complained about a dry cough. There was no respiratory-related pain but he had experienced shortness of breath during exercise for four weeks. He had not suffered from any fever or chills in the last six months. He reported occasional night sweats in the past three months.

At the age of six he had suffered from tuberculosis but had made a complete recovery. He didn’t smoke or use drugs or medication routinely. His family history was negative. The patient had no pets and had never been exposed to products that cause pulmonary damage.

On physical examination the lungs were clear on auscultation and he had normal heart sounds. His blood pressure was 130/80, his heart rate was 83 beats per minute, his O_2_ saturation was 95% and his respiratory frequency was fourteen times per minute.

There was a slight decrease in arterial pO2 (90 mmHg) with a normal pCO2 (38 mmHg).

**Figure 1:**
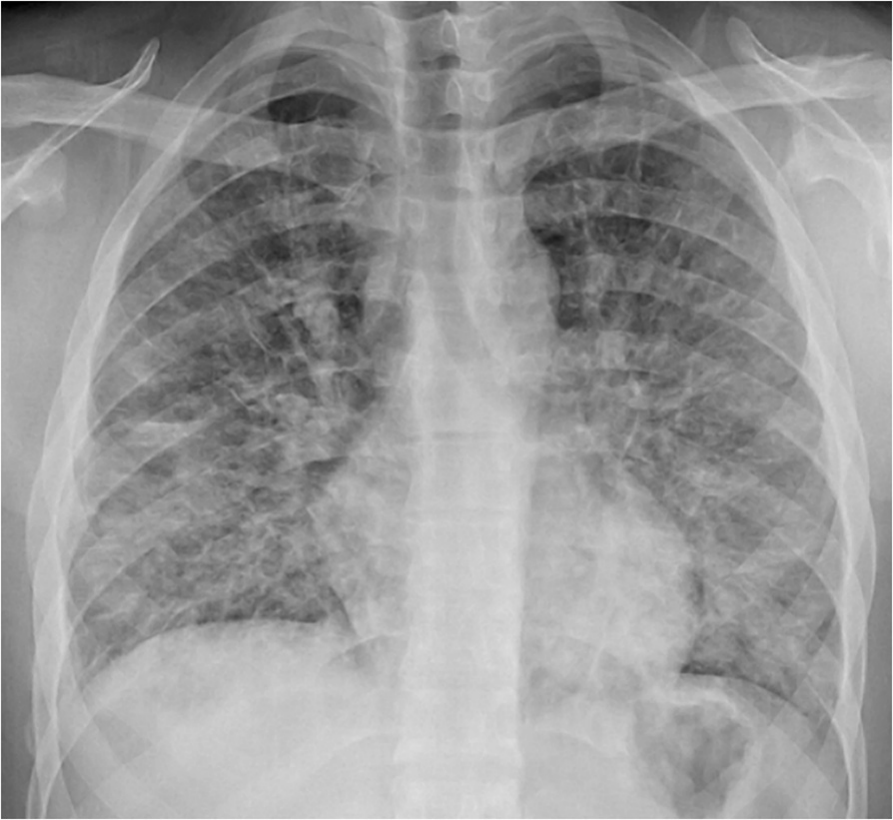
Chest X-ray at time of first admission shows diffuse diffuse reticulonodular pattern.

**Figure 2:**
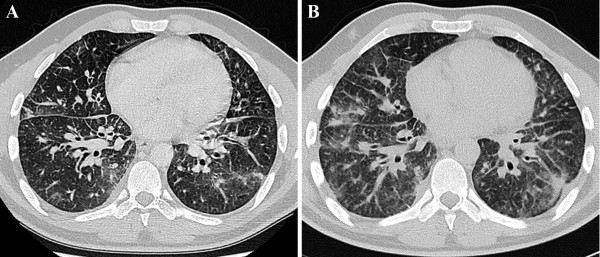
**(A) High resolution computed tomography of the chest at time of first admission shows bilateral areas of ground-glass opacification and diffusely thickened interlobular septa.** Moreover it showed the presence of hilar and mediastinal adenopathies. (**B**) Repeat scan after broad-spectrum antibiotics and high-dose corticosteroids shows progression of the interstitial disease.

Chest radiography showed a diffuse reticulonodular pattern (Figure 1). An additional computed tomography (CT) showed areas of ground-opacification and diffusely thickened interlobular septa. There were also hilar and mediastinal lymphadenopathies (Figure 2A).

Radiological evaluation was most suggestive of interstitial lung disease with a broad differential diagnosis including: infections other than tuberculosis, hypersensitivity pneumonitis, vasculitis, sarcoidosis, non-specific interstitial pneumonia, pulmonary alveolar proteinosis and lymphangitic carcinomatosis.

Biochemically there were no signs of inflammation (Table 1). An auto-immune disease was suspected so auto-antibodies were determined. Antinuclear Factor, Anti-Neutrophil Cytoplasmic Antibody, anti-Cyclic Citrullinated Protein antibodies and Rheumatoid Factor were all negative. The levels of immunoglobulins were normal and specific IgG for Aspergillus fumigatus, Alternaria alternata and Micropolyspora faeni were all negative.

**Table 1:** Blood results at first admission and in our hospital

**Test**	**First admission**	**Our hospital**	**Normal value**
Hemoglobin (g/dl)	15.3	14.0	14-18
White-cell count (x10^9/l)	8.9	17.3	4-10
Neutrophils (%)	69.6	79	38-77
Lymphocytes (%)	22.7	11.3	20-50
Platelet count (x10^9/l)	330	371	150-450
Creatinin (mg/dl)	0.75	0.73	0.7-1.3
Ureum (mg/dl)	37	35	<50
C-reactive protein (mg/l)	3.8	10.5	< 5
Prothrombin time (ratio)	1.05	1.1	0.9-1.2
Aspartate aminotransferase (U/l)	35	20	< 38
Alanine aminotransferase (U/l)	43	35	< 41
Gamma-GT (U/l)	41	27	< 53
Alkaline phosphate (U/l)	253	287	< 270
Lactate dehydrogenase (U/l)	356	302	240-480

Echocardiography showed normal function of the left ventricle with no signs of pulmonary hypertension. There was minimal pericardial effusion and no hemodynamic repercussion.

Broad-spectrum antibiotics were given for 14 days in case of eventual (atypical) infection. His clinical situation did not improve so a bronchoscopy was performed with bronchoalveolar lavage. This showed bilateral inflamed mucosa. A PCR was negative for Mycoplasma and Chlamydia. The cultures and the Ziehl-Nielsen staining were negative. PCR of the urine was negative for Legionella. Treatment was subsequently altered to high dose corticosteroids because of the suspicion of an interstitial lung disease.

As the patient developed progressive shortness of breath and became oxygen dependent, even under therapy with corticosteroids, the patient was referred to our university hospital for a second opinion. He had lost six pounds since the start of his complaints. Examination of the patient’s abdomen and heart sounds were unremarkable. We heard ronchi in the base of both lungs. There were clinically no enlarged lymph nodes, skin changes or peripheral edema.

Biochemically there was leucocytosis (17,3 x10^-9^/L compared to the normal limit: 4,3-10,0 x10^-9^/L) and C-reactive protein levels of 10,5 mg/L (normal <5 mg/L) (Table 1).

The arterial pO2 was decreased to 76 mmHg with a pCO2 of 37,5 mmHg and a pH of 7,45. The ground-glass opacities and thickening of interlobular septa at repeat CT-scan (Figure 2B) were clearly increased compared with the scan six weeks earlier. A spirometry showed a mixed obstructive restrictive pattern. Central biopsies were taken with bronchoscopy. Anatomopathological examination revealed the presence of metastatic carcinoma, probably stomach, pancreas or bile duct in origin.

An additional gastroscopy was performed. This showed an image of an extended complicated ulceration of the antrum, suspicious for malignancy. Anatomopathological evaluation of the biopsies of the ulceration at gastroscopy showed a primary adenocarcinoma of the stomach, with focal signet ring differentiation.

**Figure 3:**
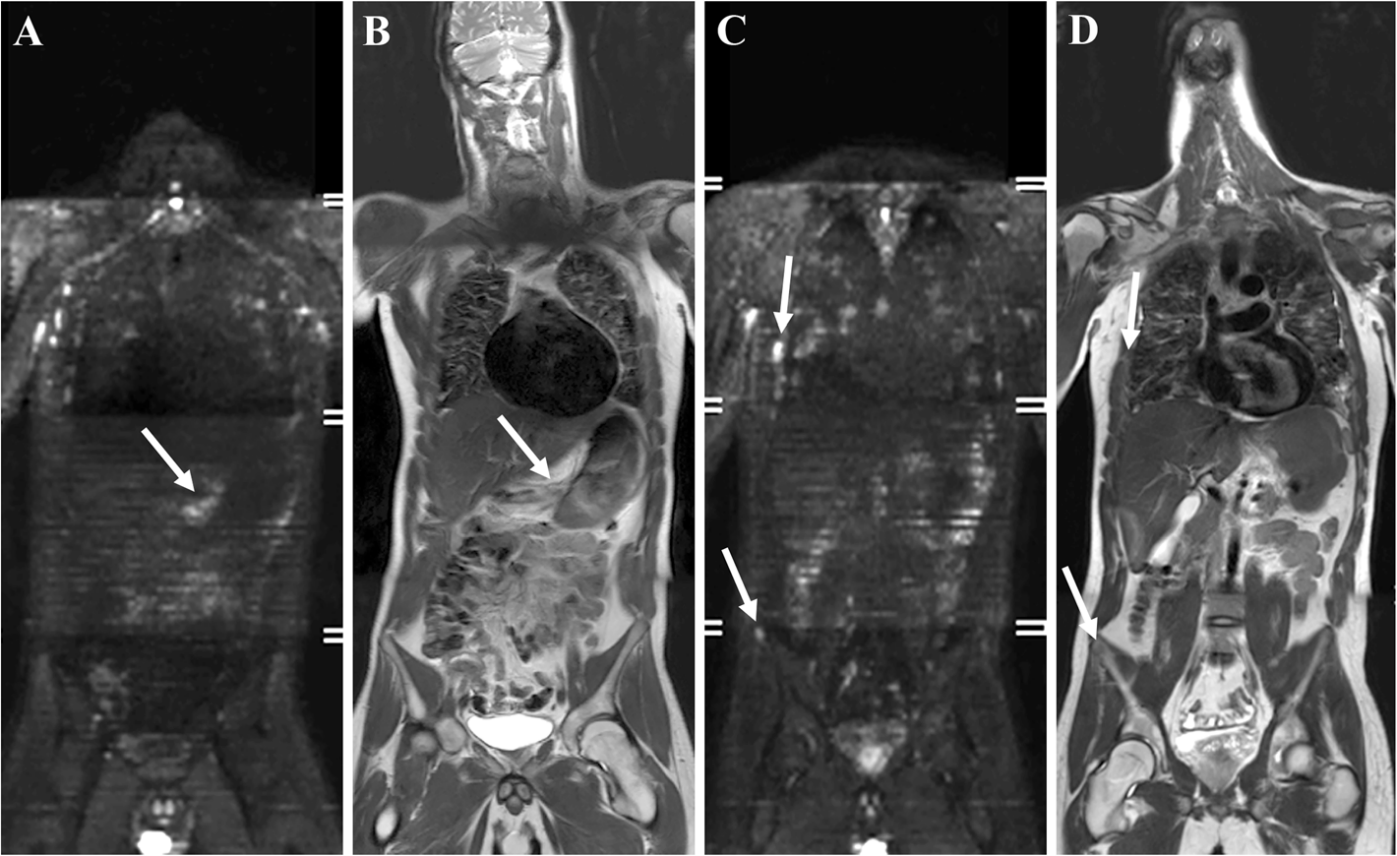
(A,B) WB-DWI with b1000-images and T2-weighted images in the coronal plane shows a tumoral mass in the lesser curvature of the stomach (arrow), (C,D) with diffuse metastases in the lungs and mediastinum as well as skeletal metastases in a right-sided rib and right iliac wing (arrows).

A whole body diffusion-weighted Magnetic Resonance Imaging (MRI) examination (WB-DWI) confirmed the presence of a large tumour at the lesser curvature of the stomach (Figure 3A, B; arrow) with diffuse lung, pleural and mediastinal metastases as well as skeletal metastases in a right-sided rib and right iliac wing, depicted as clearly hyperintense lesions on DWI-images (Figure 3C, D) . Biochemically the patient had a Carcino-embryonal antigen of 7,3 mg/L (normally <3,8 mg/L).

After establishing this final diagnosis, biweekly chemotherapy was started with cisplatinum plus 5-Fluoro-Uracil; this was well tolerated by the patient. Immediately after the start of the chemotherapy the patients’ dyspnea improved and he did not need oxygen administration.

**Figure 4:**
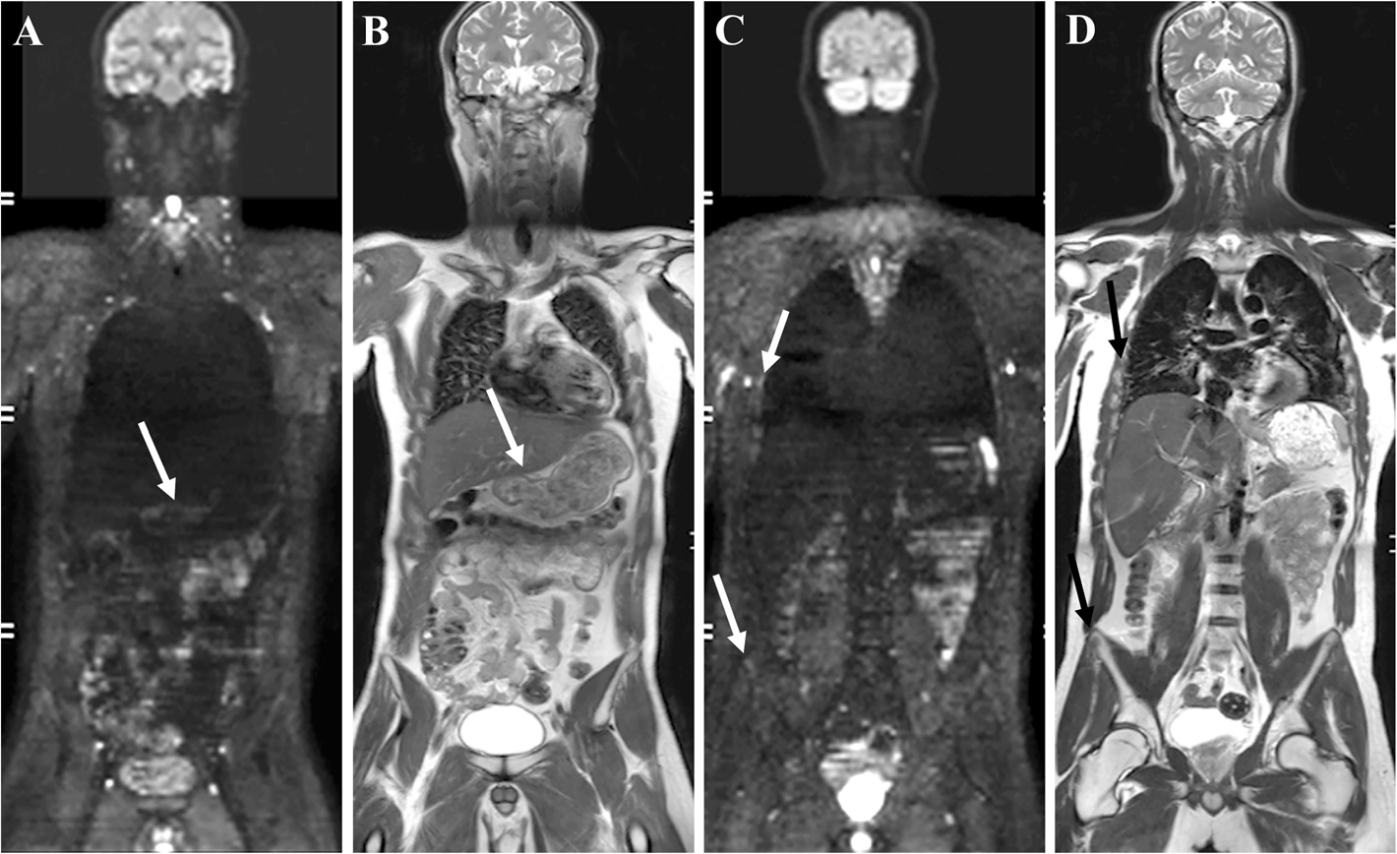
(A,B) WB-DWI with b1000-images and T2-weighted images in the coronal plane shows clear partial regression of the gastric tumour (arrow), (C,D) diffuse metastases in the lungs and mediastinum and skeletal metastases in a right-sided rib and right iliac wing (arrows).

A radiologic evaluation after 2 months of treatment revealed partial remission according to RECIST criteria, with resolution of clinical symptoms. There was clear, be it partial, regression of the primary tumour, lung, pleural, mediastinal and skeletal metastases, depicted by a clear loss of hyperintensity on DWI-images (Figure 4, arrows indicate site of gastric cancer and skeletal metastases).

## Discussion

The most common primary tumours associated with pulmonary lymphangitic carcinomatosis are breast, stomach, pancreas, lung, and prostate cancer (Table 2) [1].

**Table 2:** common locations of primary tumours associated with pulmonary lymphangitic carcinomatosis (1)

**Location**	**% of all cases**
**Breast cancer**	33
**Gastric cancer**	29
**Pancreatic cancer**	17
**Lung cancer**	4
**Prostate cancer**	3
**Other cancers**	14

This has been confirmed in a retrospective study with 43 patients with lymphangitic carcinomatosis: 23 patients had primary lung cancer, 9 breast cancer, 8 with large intestinal carcinoma and 6 gastric carcinoma [3]. However, virtually any metastatic neoplasm can cause pulmonary lymphangitic carcinomatosis, for example there are case reports where the primary tumours were of cutaneous angiosarcoma or lip cancer [4,5]. In patients with gastric cancer the lymphatic spread is most commonly directed through the diaphragm and pleural surfaces, or directed in a retrograde way through the lymphatics from hilar lymph node metastases [6].

Gastric cancer is one of the most common cancers worldwide, accounting for about eight percent of new cancers [7]. Although the worldwide incidence has declined rapidly over the recent few decades, the absolute number of new cases per year is increasing again, mainly due to aging of the world’s population. Furthermore, for reasons that are unclear, the trend towards declining incidence in young patients has been interrupted and replaced by an upward trend in recent years [8].

There are two distinct pathological types of gastric adenocarcinoma: intestinal (well-differentiated) and diffuse (undifferentiated), each with distinct morphologic appearances, pathogenesis, and genetic profiles [9]. The morphological differences are attributable to intercellular adhesion molecules, which are well preserved in intestinal-type tumours and defective in diffuse carcinomas [10]. The main carcinogenic event in diffuse adenocarcinoma of the stomach is loss of expression of E-cadherin, a key cell surface protein for establishing intercellular connections. Clinically, diffuse adenocarcinomas can give rise to infiltration of the gastric wall (i.e., linitis plastica). Diffuse type cancers are highly metastatic and characterized by rapid disease progression and a poor prognosis [11]. Signet ring histology is an independent predictor of poor prognosis in gastric adenocarcinoma [12]. The prognosis of an adenocarcinoma of the stomach is highly dependent on the stage in which the tumour is detected [13]. Accurate staging is pivotal prior in selecting appropriate therapy. MRI is emerging as an efficient whole-body imaging modality combining anatomical and functional sequences in clinically efficient scanning times with adequate spatial resolution. Several studies have indicated the additional value of WB-DWI to anatomical imaging for tumour detection, staging and (early) treatment assessment in various solid tumours [14]. WB-DWI depicts tumoral lesions by measuring water diffusion differences, which correlate with cellular density. Tumours are depicted with high signal compared to the background tissue. The resulting high contrast resolution allows for better detection of primary tumours of the gastro-intestinal tract and metastases compared to CT. In particular gastric tumours, liver metastases, lymph adenopathies, peritoneal and skeletal metastases can be detected with high accuracy [15-17].

Based on these properties and the known variable accuracy of fluorodeoxyglucose (FDG) positron emission tomography in staging gastric cancer, we opted for WB-DWI for staging and follow-up in this specific setting. Additionally, WB-DWI obviates the need for contrast-agent injection, making it useful in patients with impaired renal function or known contrast-allergy.

Patients with primary gastric cancer and lymphangitic carcinomatosis usually present with progressive shortness of breath lasting for two to four months before definite diagnosis [6]. Often these patients experience no gastric complaints at all. In a series of six young patients (aged from 21–29 years) only one had gastrointestinal symptoms (nausea and epigastric burning) [18]. A study of pulmonary function in patients with lymphangitic carcinomatosis showed a restrictive ventilatory function defect, a reduction in the diffusing capacity, reduced compliance and hypoxemia without hypercapnia [19]. Our patient had an obstructive and restrictive lung pattern, but his lung function was not well performed because of the pain.

Chest radiography and CT of gastric patients have various and often nonspecific appearances or may be normal (Table 3) [6]. Due to lack of specific radiological features, the final diagnosis can often only be made by biopsy. In the study of Dennstedt et al. the final diagnosis was only made at autopsy in four of six patients with a primary gastric tumour and lymphangitic carcinomatosis [18]. In general, diagnosis may be delayed due to the combination of aspecific symptoms, uncertain initial diagnostic work-up and a low index of suspicion for malignancy.

**Table 3:** Radiologic characteristics of pulmonary lymphangitic carcinomatosis (6)

	
**Chest radiography**	
- Coarse bronchovascular markings with irregular outline	
- Coarse reticulonodular pattern with intraparenchymal extension of tumour	
- Unilateral or bilateral changes predominantly in the lower lobes of the lungs	
- Kerley A and B lines	
- Hilar and mediastinal lymphadenopathy (20-40% of cases); usually asymmetric	
- Pleural effusion (30-50% of cases)	
- No abnormalities (30-50% of cases)	
**Chest CT scan**	
- Smooth (early stage) and nodular (late stage) thickening of interlobular septa and peribronchovascular interstitium	
- Polygonal arcades with thickened limbs from thickened septa of adjacent lobules	
- Normal lung architecture is maintained	
- Ground-glass appearance from interstitial edema or extension of the tumour into the parenchyma	

The general prognosis of patients with lymphangitis carcinomatosis is poor with an average survival of only three months in historical series [1]. Patients included in the study of Dennstedt et al. (six patients with an average age of 26 years and a primary gastric tumour) had a mean survival time of only 22 days after their first admission to hospital [14]. Anatomopathological examination revealed an adenocarcinoma of the diffuse type in all of the patients [18]. Moreover, both the patient in the study of Desigan [6] and our patient had an adenocarcinoma with focal signet cell carcinoma, which is associated with a worse prognosis [12,20], although there are publications that didn’t show a prognostic effect [21].

We might therefore conclude that an adenocarcinoma of the diffuse type might be a predictor for lymphangitic carcinomatosis. The number of patients is too small to make definitive conclusions.

## Conclusion

It is important to consider the possibility of lymphangitic carcinomatosis in patients with: progressive dyspnoea; no other known causes of interstitial lung disease; or an interstitial pattern demonstrated on CT or chest radiographs. When there is doubt about the exact diagnosis, a biopsy must be performed in order not to lose time for starting the necessary treatment, even in young patients where a malignancy is not expected.

## Consent

Written informed consent for publication of the clinical details and clinical images was obtained from the patient.

## Competing interests

The authors declare that they have no competing interests.

## Authors’ contributions

HP and KM designed the study. HP, WW, KM and VV drafted and edited the manuscript. HP, KM and WW treated and observed the patient including follow-up. VV acquired the radiographic pictures. All authors read and approved the final manuscript.
